# Supplementary material for: Variants rs3804099 and rs3804100 in the TLR2 Gene Induce Different Profiles of TLR-2 Expression and Cytokines in Response to Spike of SARS-CoV-2
Source: Int J Mol Sci. 2024 Oct 15;25(20):11063. doi: 10.3390/ijms252011063 (PMC11507191; doi:10.3390/ijms252011063)
Supplement: Supplementary file 1 [file ijms-25-11063-s001.zip › ijms-3257092-supplementary.pdf]

Supplementary Table S1. Allele and genotype frequencies of SNPs in *TLR2*

Genotypes/ Alleles	ARDS severe n=413, %	ARDS mild-moderate n=605, %	p-value
rs3804099			
TT	49.6	52.2	
TC	43.5	41.6	0.715
CC	6.9	6.1	
T	71.3	73.1	0.460
C	28.6	26.9	
rs3804100			
TT	87.1	86.6	
TC	12.1	12.8	0.950
CC	0.7	0.6	
T	93.2	92.9	0.896
C	6.8	7.1	

ARDS: Acute Respiratory Distress Syndrome.

Supplementary Table S2. Haplotype analysis of SNPs in *TLR2*.

Haplotype rs3804099-rs3804100	ARDS severe n=413, %	ARDS mild-moderate n=605, %	p-value
T-T	71.0	72.8	0.376
C-T	22.2	20.0	0.245
C-C	6.5	6.8	0.795

Supplementary Table S3. Genotypes of patients selected for *in vitro* assay

ID	rs3804099	rs3804100
COV418	TT	TT
COV794	TT	TT
COV454	TT	TT
COV500	T**C**	T**C**
COV486	TT	TT
COV421	T**C**	T**C**
COV809	TT	TT
COV819	TT	TT
COV481	TT	TT
COV485	T**C**	TT
COV523	**CC**	TT
COV610	TT	TT

Supplementary Table S4. Antibodies used for flow cytometry

Antibody	Conjugate to	Clone[1]/Catalog
CD3	APC-Cy7	HIT3a
CD4	PerCP	NA
CD8	BV510	SK1
CD14	PerCP	HCD14
CD19	FITC	HIB19
TLR-2	Pe-Cy7	TL2.1
HLA-DR	APC	L243
Viability	PE-TexRed	NA
CD69	PE	FN50
CD56	APC	5.1H11
CD27	PerCP	O323
IgM	APC-Cy7	MHM-88
IgD	BV510	IA6-2
Human CD8/NK Panel	NA	740267

1 The clone is indicated for flow cytometry antibodies, and the catalog number for the LegendPlex kit. NA, not applicable. Antibodies were of Biolegend Company, except CD4, which was of BD Bioscience

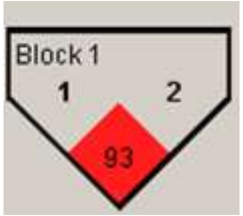

Supplementary Figure S1. Analysis of linkage disequilibrium of rs3804099-rs3804100 (*TLR2*) in the study population.

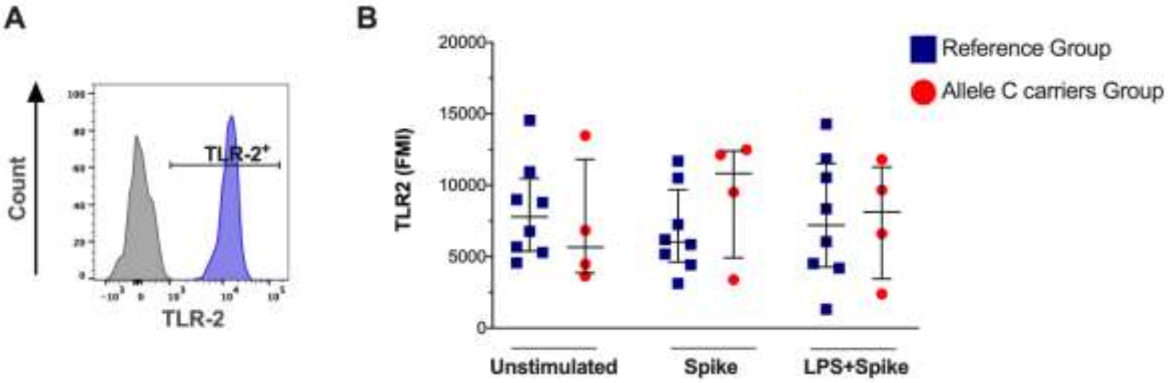

Supplementary Figure S2. TLR-2 expression in monocytes. PBMCs from two groups of patients with TT genotype (rs3804099 and rs3804100) and carriers of C allele in at one SNP (*TLR2*) were stimulated for 24 hours with the spike protein (1µg/mL) or spike + LPS (1µg/mL). An unstimulated condition was included as a control stimulation (Unstimulated). (**A**) Flow cytometry histogram of TLR-2 (blue) expression on monocytes. Black shadow: FMO control. (**B**) Analysis of TLR-2 expression on monocytes. Data are represented as median and IQR values. The Kruskal-Wallis test performed statistical comparisons.

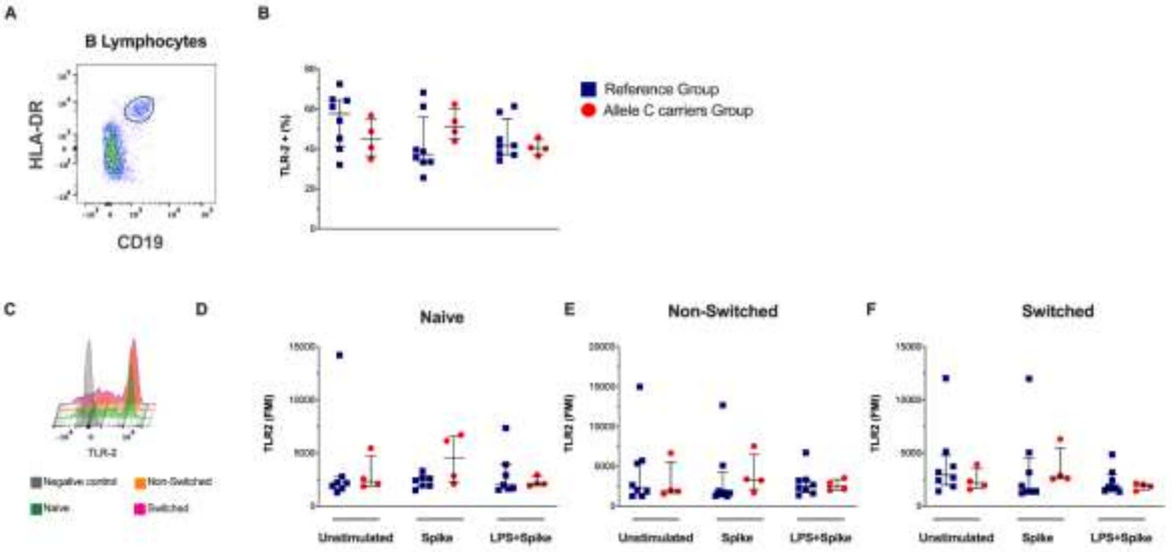

Supplementary Figure S3. TLR-2 B-cell frequency. PBMCs from two groups of patients with TT genotype in rs3804099 and rs3804100 and carriers of C allele in at one SNP (*TLR2*) were stimulated for 24 hours with Spike (1µg/mL) or spike + LPS (1µg/mL each one). An unstimulated condition was included as a control stimulation (Unstimulated). **A**) A representative dot plot shows the CD19 and HLA-DR markers gated the B lymphocyte distribution. (**B**) Analysis of B-cell TLR-2+ frequencies. (**C**) Flow cytometry histograms of TLR-2 compared between B-cells subsets. Black shadow: FMO control. Analysis of TLR-2+ frequencies in (**D**) naïve, (**E**) Non-switched, and (**F**) switched. Data are represented as median and IQR values. The Kruskal-Wallis test performed statistical comparisons.

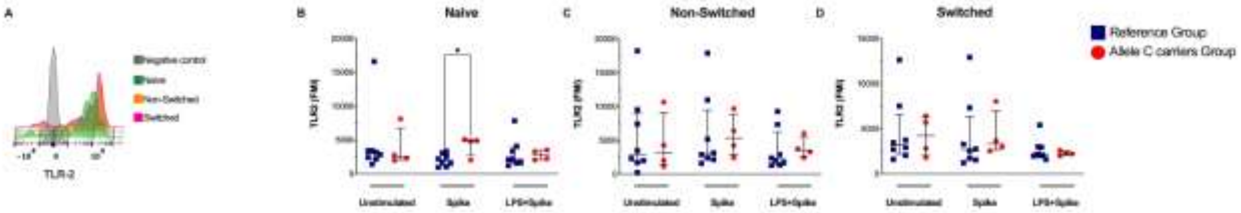

Supplementary Figure S4. TLR-2 frequency on activated B cells. PBMCs from two groups of patients with TT genotype in rs3804099 and rs3804100 and carriers of C allele in at one SNP (*TLR2*) were stimulated for 24 hours with the spike protein (1µg/mL) or spike + LPS (1µg/mL each one). An unstimulated condition was included as a control stimulation (Unstimulated). Cells were analyzed by flow cytometry. (**A**) Flow cytometry histograms of TLR-2 compared between activated B cell subsets. Black shadow: FMO control. Analysis of TLR-2 expression in activated (**B**) naïve, (**C**) Non-switched, and (**D**) switched. Data are represented as median and IQR values. The Kruskal-Wallis test performed statistical comparisons.

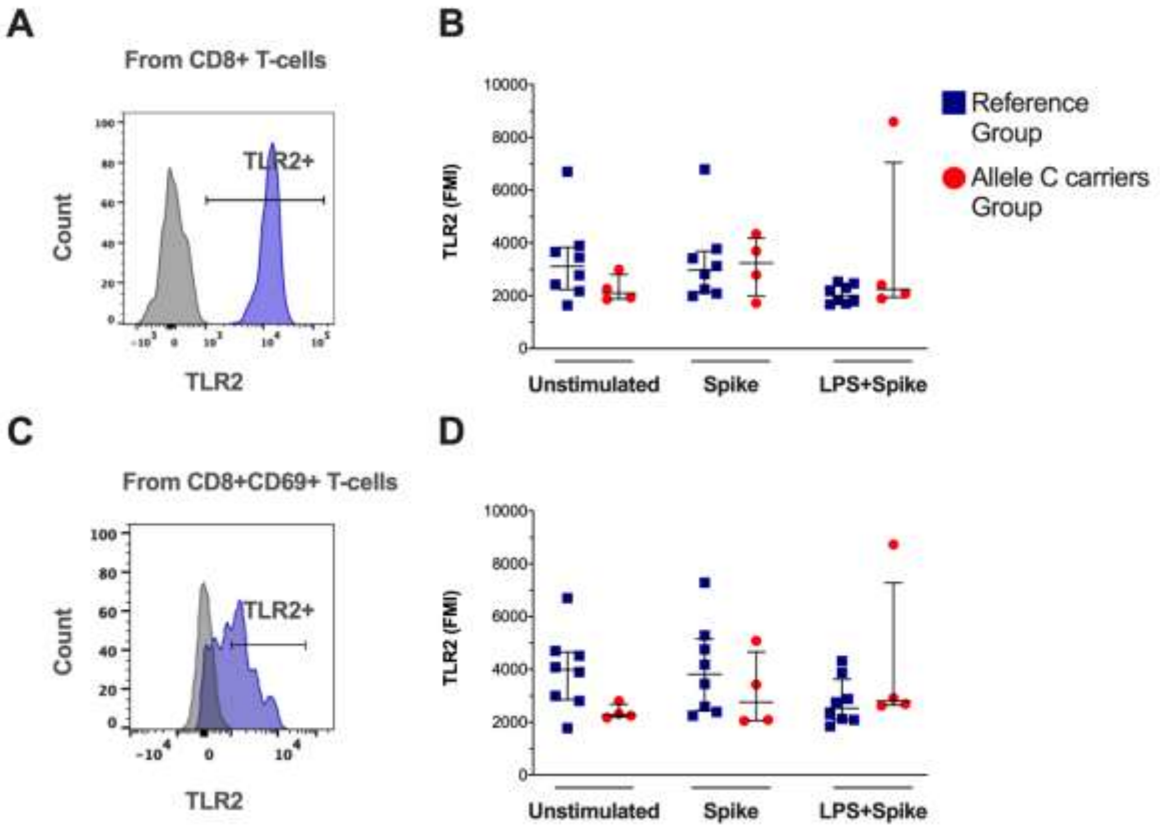

Supplementary Figure S5. TLR-2 frequency on CD8+ T cells. Cells from two groups of patients with TT genotype in rs3804099 and rs3804100 and carriers of C allele in at one SNP (*TLR2*) were stimulated for 24 hours with the spike protein (1µg/mL) or spike + LPS (1µg/mL each one). An unstimulated condition was included as a control stimulation (Unstimulated). (**A**) Flow cytometry histogram of TLR-2 (blue) expression on CD8+ T-cells. Black shadow: FMO control. Analysis of TLR-2 expression in (**B**) CD8+ T-cells. (**C**) Flow cytometry histogram of TLR-2 (blue) expression on CD8+CD69+ T-cells. Black shadow: FMO control. Analysis of TLR-2 expression in (**D**) CD8+CD69+ T-cells. Data are represented as median and IQR values. The Kruskal-Wallis test performed statistical comparisons.

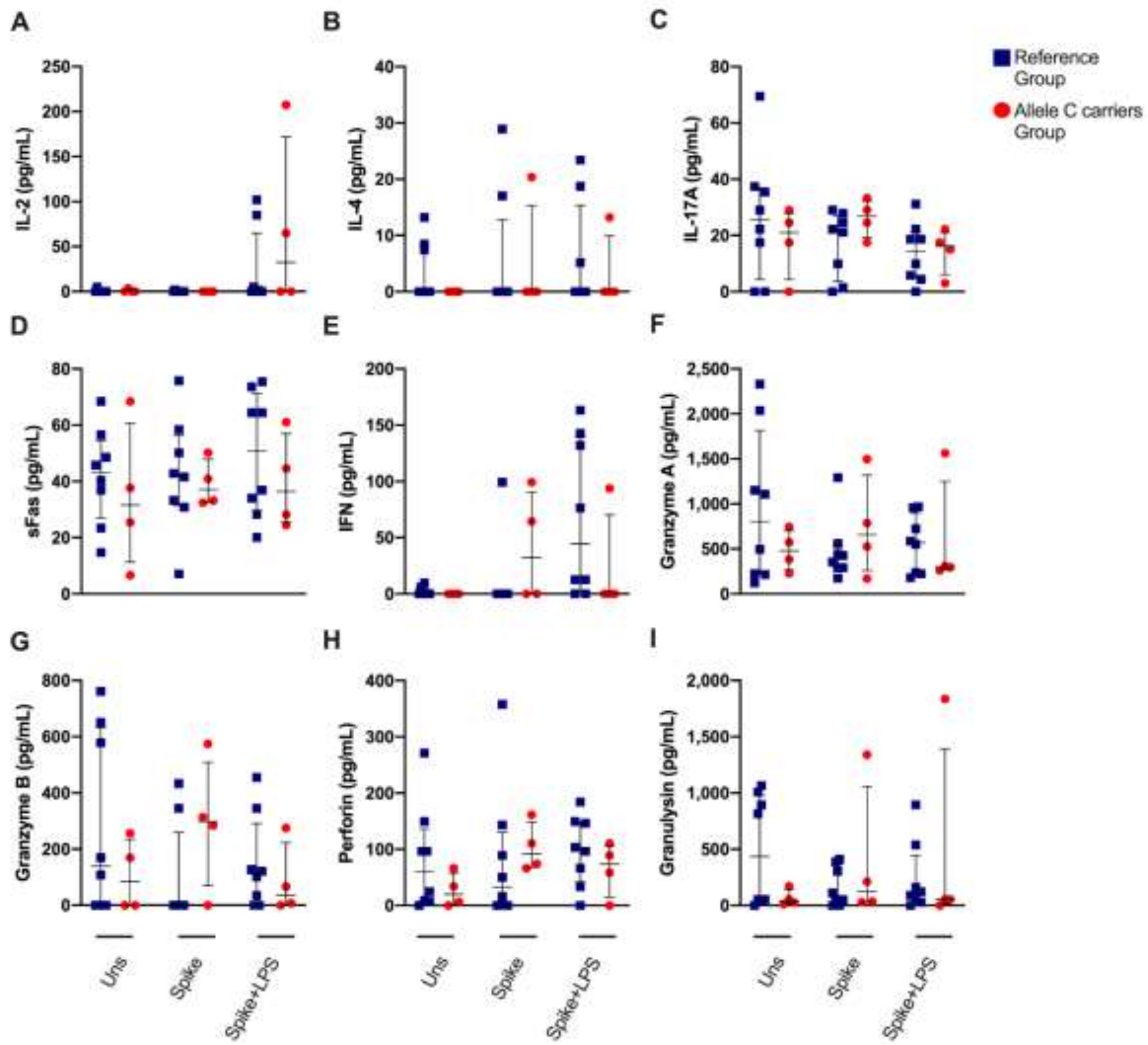

Supplementary Figure S6. Protein levels into stimulated culture supernatants. Mononuclear cells from two groups of patients with TT genotype in rs3804099 and rs3804100 and carriers of C allele in at one SNP (*TLR2*) were stimulated for 24 hours with spike protein (1µg/mL) or spike + LPS (1µg/mL each one). An unstimulated condition was included as a control stimulation (Unstimulated). The supernatant was recovered to measure a cytotoxic protein panel by flow cytometry. Quantitative analysis of (**A**) IL-2, (**B**) IL-4, (**C**) IL-17A, (**D**) sFas, (**E**) IFN, (**F**) Granzyme A, (**G**) Granzyme B, (**H**) Perforin, and (**I**) Granulysin levels. Data are represented as median and IQR values. The Kruskal-Wallis test performed statistical comparisons.

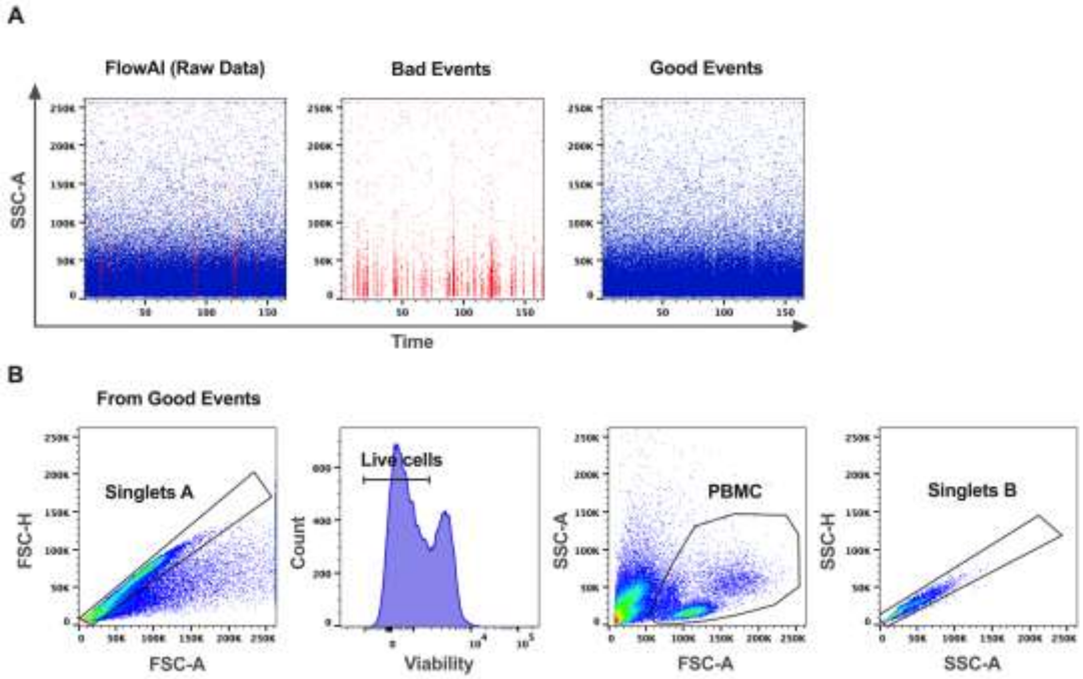

Supplementary Figure S7. Representative results were obtained in PBMCs from two groups, including patients with TT genotype in rs3804099 and rs3804100 and carriers of C allele in at least one SNP (*TLR2*). (**A**) A quality control analysis using flowAI was performed to detect and remove anomalies from FCS data. After flowAI analysis, the analysis strategy consisted of limiting the singlet cells through forward scatter (FSC-A versus FSC-H) and side scatter (SSC-A versus SSC-H). (**B**) Next, viability plots were selected. Then, PBMC was set through FSC-A versus SSC-A to identify monocytes, B lymphocytes, and cytotoxic cells, including CD8 T-cell, NK and NKT cells, and CD4 T-lymphocytes through CD molecules previously mentioned and summarized in the results section.
